# Modular Hub Genes in DNA Microarray Suggest Potential Signaling Pathway Interconnectivity in Various Glioma Grades

**DOI:** 10.3390/biology13040206

**Published:** 2024-03-23

**Authors:** Marco A. Orda, Peter Matthew Paul T. Fowler, Lemmuel L. Tayo

**Affiliations:** 1 School of Chemical, Biological, and Materials Engineering and Sciences, Mapúa University, Manila City 1002, Philippines; maorda@mymail.mapua.edu.ph (M.A.O.); pmptfowler@mapua.edu.ph (P.M.P.T.F.); 2 School of Graduate Studies, Mapúa University, Manila City 1002, Philippines; 3 Department of Biology, School of Health Sciences, Mapúa University, Makati City 1203, Philippines

**Keywords:** glioma, WGCNA, PI3K/Akt pathway, drug repurposing, progesterone

## Abstract

**Simple Summary:**

Currently, gliomas still stand as one of the global health concerns due to its resistance to treatment strategies, resulting in poor survival rate and high mortality despite sustained efforts. Even though chemotherapeutic and targeted drugs for glioma exist, its heterogeneity per grade and cell origin poses as a significant contributing factor to its negative response to treatment. With this, our study utilized a systems biology approach to investigate correlations between various grades of glioma using DNA microarray profiles of samples that contain pilocytic astrocytoma, oligodendroglioma, anaplastic astrocytoma, and glioblastoma multiforme gene expression data. The highly preserved gene clusters (modules) and their corresponding hub genes were identified, which provided further insights into the signaling pathways and cellular processes involved. Drug repurposing was also performed based on the upregulated and downregulated hub genes. This approach determined potential drug candidates that are known to have mediating effects on the signaling pathways, thereby highlighting the possibility of the gene network as a potential therapeutic avenue.

**Abstract:**

Gliomas have displayed significant challenges in oncology due to their high degree of invasiveness, recurrence, and resistance to treatment strategies. In this work, the key hub genes mainly associated with different grades of glioma, which were represented by pilocytic astrocytoma (PA), oligodendroglioma (OG), anaplastic astrocytoma (AA), and glioblastoma multiforme (GBM), were identified through weighted gene co-expression network analysis (WGCNA) of microarray datasets retrieved from the Gene Expression Omnibus (GEO) database. Through this, four highly correlated modules were observed to be present across the PA (GSE50161), OG (GSE4290), AA (GSE43378), and GBM (GSE36245) datasets. The functional annotation and pathway enrichment analysis done through the Database for Annotation, Visualization, and Integrated Discovery (DAVID) showed that the modules and hub genes identified were mainly involved in signal transduction, transcription regulation, and protein binding, which collectively deregulate several signaling pathways, mainly PI3K/Akt and metabolic pathways. The involvement of several hub genes primarily linked to other signaling pathways, including the cAMP, MAPK/ERK, Wnt/β-catenin, and calcium signaling pathways, indicates potential interconnectivity and influence on the PI3K/Akt pathway and, subsequently, glioma severity. The Drug Repurposing Encyclopedia (DRE) was used to screen for potential drugs based on the up- and downregulated hub genes, wherein the synthetic progestin hormones norgestimate and ethisterone were the top drug candidates. This shows the potential neuroprotective effect of progesterone against glioma due to its influence on EGFR expression and other signaling pathways. Aside from these, several experimental and approved drug candidates were also identified, which include an adrenergic receptor antagonist, a PPAR-γ receptor agonist, a CDK inhibitor, a sodium channel blocker, a bradykinin receptor antagonist, and a dopamine receptor agonist, which further highlights the gene network as a potential therapeutic avenue for glioma.

## 1. Introduction

Glioma is a type of brain tumor that originates from glial cells. A common type of glioma, astrocytoma, emerges from astrocytes, cells that primarily provide structural support to neurons and regulate the blood–brain barrier. Astrocytomas are often considered to be more aggressive than other types of glioma due to their high cell proliferation, invasiveness, and recurrence [1]. Astrocytomas can be further classified based on WHO grades of increasing malignancy: Grade I: pilocytic astrocytoma (PA); Grade II: diffuse astrocytoma (DA); Grade III: anaplastic astrocytoma (AA); and Grade IV: glioblastoma multiforme (GBM) [2]. On the other hand, oligodendrogliomas are less-common gliomas that originate from oligodendrocytes, which produce and maintain the myelin sheath around the neuronal axons for nerve signal transmission. Like astrocytomas, they are classified based on WHO grades, wherein oligodendroglioma (OG) is Grade II and anaplastic oligodendroglioma (AO) is Grade III [3]. They are noted for their specific genetic alterations that affect their prognosis and treatment, specifically *1p*/*19q* codeletion, as these alterations are usually present in both grades; in contrast, different grades of astrocytomas contain key sets of genetic alterations [2,4]. Despite their differences, astrocytomas and oligodendrogliomas may share similar genetic alterations and clinical symptoms [5].

Along with surgery and radiation therapy, chemotherapeutic and targeted drugs, particularly temozolomide and bevacizumab, are used as treatment for glioma [6]. Several studies have also indicated the use of inhibitors for the epidermal growth factor receptor (EGFR) due to the strong involvement of the downstream PI3K/Akt pathway in glioma cell proliferation [7,8]. However, resistance against temozolomide and the toxicity of bevacizumab have been documented [9,10], while EGFR inhibitors tend to struggle in penetrating the blood–brain barrier [11]. Hence, the poor prognosis of the five-year survival rate of patients with GBM has not improved significantly despite sustained efforts [12,13]. As a result, systems biology approaches, which excel in identifying gene network hubs that play central roles in regulating overall cellular processes, are currently being considered. One of these approaches is the weighted gene co-expression network analysis (WGCNA), which is used to characterize the patterns of gene association found in the gene expression data of several diseases. This method enables identifying potential therapeutic targets linked to particular biological processes or illnesses by categorizing genes into modules according to their expression profiles, offering a new point of view for the comprehensive understanding of the molecular foundations of related diseases. Furthermore, the identification of hub genes in the modules can also be done to provide insight into the main pathways and processes that are mutated. Lastly, these hub genes can then be subjected to drug repurposing techniques, which has emerged as a novel approach in the field of drug discovery due to its potential to save time and costs, as it enables the screening of approved and experimental drugs that can interfere with the mutated genes.

In this study, gene expression datasets for four different grades of glioma, represented by PA (GSE50161), OG (GSE4290), AA (GSE43378), and GBM (GSE36245), were acquired from the Gene Expression Omnibus (GEO) and were then used to perform a meta-analysis approach through WGCNA to determine highly conserved modules among all the datasets. Four such modules were identified and then subjected to functional annotation and pathway enrichment analysis, where it was found that they were highly involved in signal transduction, transcription regulation, protein binding, and metabolic changes. In each module, the protein–protein association network and key hub genes were determined, which further allowed for screening of potential already-existing drugs. With this bioinformatics approach, this study aims to offer further insights for understanding the pivotal points at which different glioma grades align genetically to pave the way for the enhancement of existing treatment strategies or the development of novel ones.

## 2. Materials and Methods

### 2.1. Dataset Acquisition and Preparation

The microarray gene expression datasets were sourced from the National Center for Biotechnology Information: Gene Expression Omnibus (NCBI GEO) [https://www.ncbi.nlm.nih.gov/geo/ accessed on 7 December 2023] online database for WGCNA analysis. The datasets gathered contained expression data taken from primary tumor samples of patients diagnosed with pilocytic astrocytoma (PA), oligodendroglioma (OG), anaplastic astrocytoma (AA), and glioblastoma multiforme (GBM). Furthermore, only the datasets carried out through GPL570-HG-U133 Plus 2 Affymetrix Human Genome U133 Plus 2.0 Array were selected in order to prevent inconsistencies arising from probe design, normalization issues, and batch effects [14,15,16]. This initial screening resulted in the exclusion of datasets for diffuse astrocytoma (DA) and anaplastic oligodendroglioma (AO) from further investigation. A total of 113 samples were obtained. Table 1 summarizes the details and distribution of samples for the four tumor types.

All screened raw data underwent background correction, quantile normalization, and log-2 transformation through the robust multi-array average (RMA) method in the affy package in Bioconductor using R [http://www.bioconductor.org accessed on 19 December 2023]. A boxplot of expression values and sample clustering dendrograms were generated to visually inspect the resulting data for outliers. After pre-processing, control probes were removed to eliminate non-biological variation and focus on the samples containing primary tumor expression. Then, the expression data were filtered to select the genes whose mean and variance throughout all the samples in each dataset were higher than the 20% percentile cut-off. Probe IDs were converted into gene symbols through the AnnotationDbi function using the hgu133plus2.db database for biological interpretation and analysis. Lastly, only the probes that were present in all datasets were used, and samples with no values after log-2 transformation were filtered out using the WGCNA R package’s goodSamplesGenesMs function.

### 2.2. Weighted Gene Co-Expression Network Analysis (WGCNA)

#### 2.2.1. Scale-Free Network Approximation

Using the pickSoftThreshold function of the WGCNA R package, a plot of the scale-free topology fit versus power index (1–20) was generated. The soft-thresholding power (β) was then selected as the lowest power for which the scale-free topology criterion is satisfied. In a scale-free topology, the distribution of gene degrees (number of gene connections) follows the power-law distribution. This is often evaluated based on how well the co-expression network conforms to the linear relationship between log of connectivity and log of connectivity probability—by plotting the scale-free topology fit versus soft-thresholding power and selecting the power at which the scale-free topology fit reaches a plateau or a reasonably high value that indicates a good fit [17]. Hence, an approximate straight-line relationship was plotted using the values for soft connectivity (k) to evaluate the chosen soft-thresholding power.

#### 2.2.2. TOM-Based Network Construction and Module Identification

Using Pearson’s correlation with the network type “signed,” the adjacency matrices were generated for network construction. The Topological Overlap Measure (TOM) dissimilarities were then calculated using the soft-thresholding power to raise the adjacency matrices. This allowed the emphasis of the strong correlations and the downweighting of weak correlations in performing fast hierarchal clustering of genes using the flashClust function. To produce gene dendrograms using the highly correlated genes, the hclust function was used to perform hierarchal clustering based on the distance matrix of the expression profiles with the “average” method. Gene modules were identified based on hierarchal clustering through the cutreeHybrid function, in which the deep split parameter was tested from 0 to 3 for branch splitting sensitivity [18]. Stable modules that remain consistent across different values of a deep split parameter indicate that the clustering algorithm has accurately identified significant clusters within the datasets, while varying results would suggest additional validation of the parameters used, such as the soft-thresholding power.

#### 2.2.3. Module Preservation Analysis

Module preservation analysis measures the extent to which the connectivity patterns of the reference network are preserved in other networks to evaluate the biological relevance of the gene modules across different diseases or disease states. The modulepreservation function from the WGCNA R package was used to analyze gene co-expression network preservation of PA, OG, AA, and GBM modules qualitatively and quantitatively with the network type “signed,” the number of permutations set to 100, and a minimum module size of 30 genes. After then, the eigengene-based connectivity (kME), a measure of the correlation between the expression profile of each gene and its gene co-expression network, was calculated to identify the genes within each module using the moduleEigengenes function. Module membership was associated to the correlation between the gene expression profile and its module eigengene.

### 2.3. Functional Annotation and Pathway Enrichment

The Database for Annotation, Visualization, and Integrated Discovery (DAVID) was used to perform functional annotation clustering on the modules of interest [19]. Within DAVID Gene Ontology (GO), the databases for biological processes (BP), cellular components (CC), and molecular functions (MF) were utilized. This database contains information on the basic features and activities of the provided genes based on the proteins they encode. The classification stringency was set to medium, and only significant GO terms (*p* adj. < 0.05) that have enrichment scores greater than 1.3 were chosen for analysis. For pathway enrichment analysis, searches through the Kyoto Encyclopedia of Genes and Genomes (KEGG) were performed. KEGG terms that scored significantly and clustered with the selected GO terms were then used to provide details on the overall functions of the modules of interest [20].

### 2.4. Identification of Protein-Protein Interaction (PPI) and Hub Genes

In generating PPI networks, genes within each of the highly preserved modules were considered for potential associated protein–protein interactions through the Search Tool for the Retrieval of Interacting Genes/Proteins (STRING) database [21]. PPI networks with a minimum interaction score of 0.7 (high confidence) were built for each module of interest. The generated networks were then imported into Cytoscape to find hub genes based on degree centrality using the Cytohubba feature, which measures the number of interactions that the gene has within the network that correlates with its essentiality [22,23]. In this case, the top 10 genes with the highest interactions within the modules were considered hub genes.

### 2.5. Signature-Based Drug Repurposing

The top 10 hub genes identified based on their degree centrality were first classified as either being “upregulated” or “downregulated” using GEO2R [https://www.ncbi.nlm.nih.gov/geo/geo2r/ accessed on 23 December 2023] prior to searching for potential drugs. The Drug Repurposing Encyclopedia (DRE) webserver was used to search for available drugs that can be repurposed based on their transcription profiles from the Molecular Signatures Database (MSigDB) and Connectivity Map (CMap) [24,25]. The groups of upregulated and downregulated genes were submitted for drug repurposing tests based on molecular signature screening. Experimental drugs without an indicated mechanism of action were excluded from the analysis, and only candidates with false discovery rates (FDR) of less than 0.05 were considered.

## 3. Results

### 3.1. Weighted Gene Co-Expression Network Analysis (WGCNA)

#### 3.1.1. Data Pre-Processing and Approximation of Scale-Free Networks

**Figure 1:**
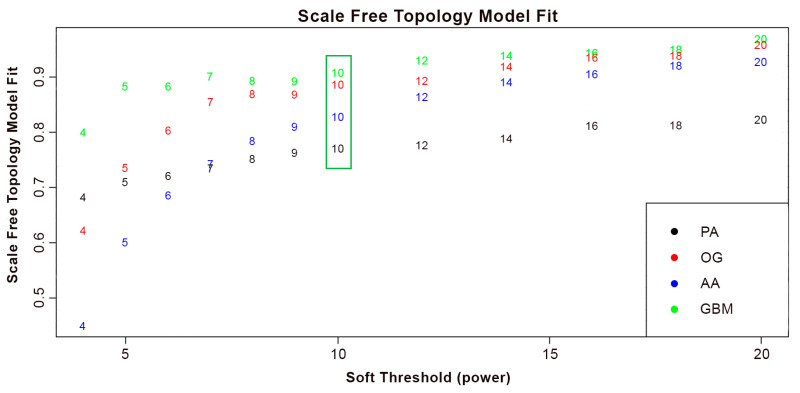
Network indices to approximate scale-free topology through average number of connections per gene in the networks. Numbers represent the scale free topology model fit per dataset at given soft-thresholding power. The green box indicates the point at which the index across the datasets has stabilized (β = 10).

**Figure A1:**
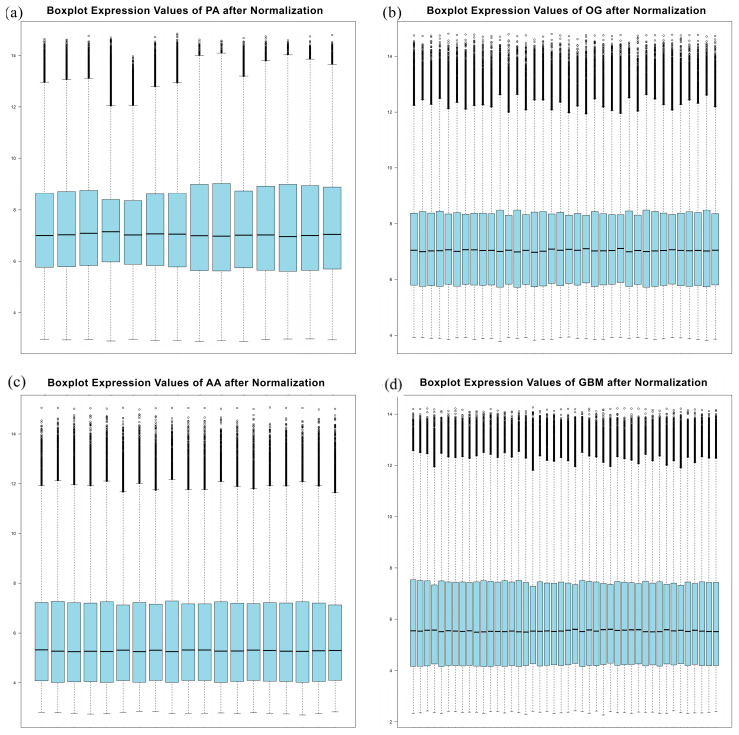
Boxplot of microarray datasets (**a**) PA, (**b**) OG, and (**c**) AA and (**d**) GBM datasets after data preparation and filtering.

**Figure A2:**
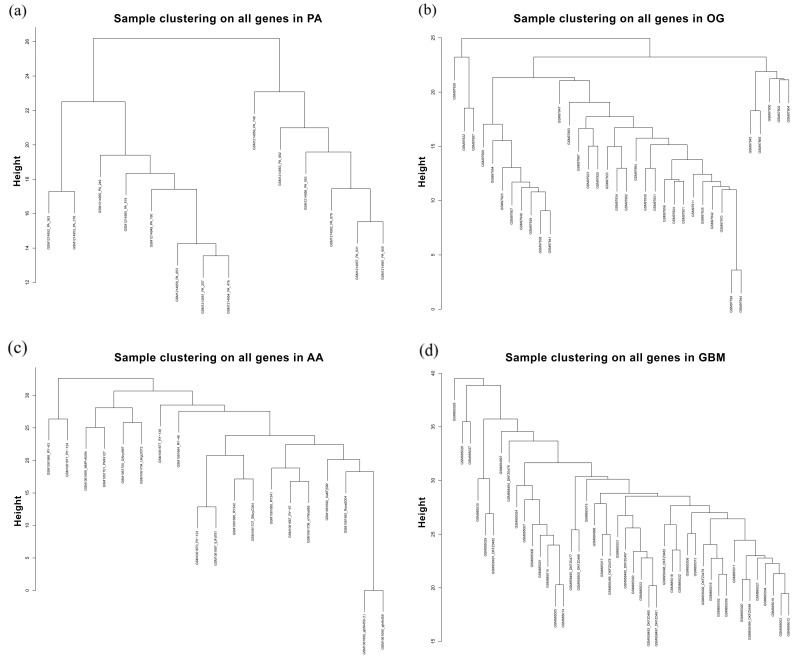
Sample clustering dendrogram of (**a**) PA, (**b**) OG, (**c**) AA, and (**d**) GBM datasets. The ends of each branch represent the individual samples in the datasets.

After the data preparation and filtering stages (Figure A1), a total of 25,466 genes remained. All samples for each dataset were included in the weighted gene co-expression network analysis, as no significant outliers were identified based on the sample clustering dendrograms of the datasets in Figure A2. Figure 1 shows the variation of the scale-free topology fit index versus the soft threshold (β) values ranging from 1 to 20. At β > 10, it can be observed that the index across all the datasets has stabilized and has steadily plateaued onwards. This indicates that the scale-free topology fit for β > 10 is not any more significantly affected by the increase in power and that a robust scale-free structure has been achieved. With this, it was decided that the scale-free topologies for all datasets would be approximated as β = 10. The approximation of the optimal soft-thresholding power for the scale-free topology fit is crucial, as it guarantees robustness, efficiency, and resilience to errors of the gene network. Lower soft-thresholding power leads to denser gene networks with more connections, supporting the selection of the least power at which the network indices have stabilized. This ensures that the resulting modules will contain biologically relevant relationships between genes.

**Figure 2:**
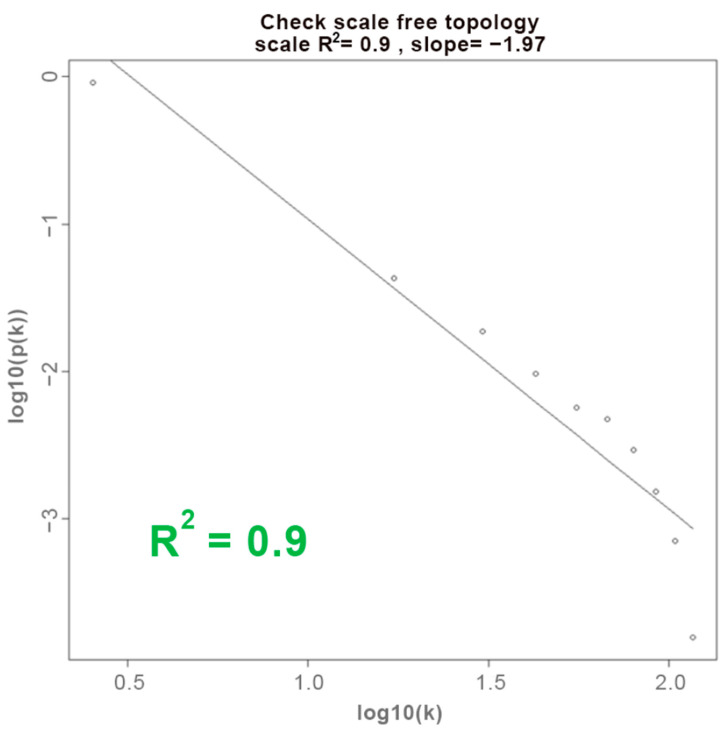
Approximate linear relationship for the GBM dataset at β = 10 presented by log-log plot.

Figure 2 shows the corresponding log-log plot for the GBM dataset, constructed by plotting the connectivity frequencies corresponding to each value of connectivity at a soft-thresholding power of 10. This dataset exhibited the most accurate representation of a scale-free network by achieving the highest R2 value of 0.9 among the datasets, which suggests that gene co-expression networks derived from this dataset are more likely to have related expression patterns, features, or information for confident interpretation of the network regarding its biological significance. Hence, the GBM dataset was used as the reference dataset for WGCNA.

#### 3.1.2. TOM-Based Network Construction and Module Identification

**Figure 3:**
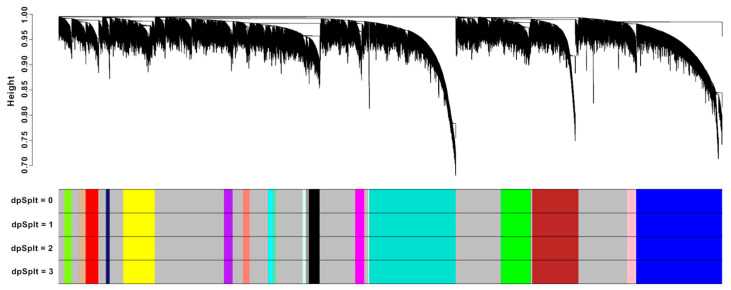
Dendrogram of gene clustering on TOM-based dissimilarity and module split sensitivities of GBM dataset. The assorted colors represent identified gene co-expression modules corresponding to the portion of the dendrogram directly above.

Several approaches to conducting meta-analysis through WGCNA exist, one of which involves designating a single dataset as a reference and then projecting the module eigengenes of other datasets onto this reference. The choice of the reference dataset plays a crucial role in determining the network’s robustness, considering factors such as the dataset’s sample size, scale-free network approximation, and the resolution of clustering in the gene dendrograms based on the TOM gene dendrograms [22]. Following these criteria, the GBM dataset was chosen as the reference for the meta-analysis, and the identified modules are shown in Figure 3. Aside from this, it aligns with the fact that GBM is the end stage of glioma, which makes it suitable to be referenced to glioma datasets of lower grades, as it may indicate modules that are involved in glioma progression from the initial and latter stages. It can be observed from the sensitivity plots that the same sets of modules remained despite the increasing sensitivity, which may be due to strong correlation of the genes, having fewer significant gene clusters, or both (Figure S1). All in all, WGCNA identified a total of 21 gene co-expression modules classified arbitrarily by different colors: green-yellow (270), gold (100), red (492), midnight blue (140), yellow (1215), purple (327), tan (265), cyan (180), light green (80), light cyan (119), black (409), magenta (351), pink (323), gray60 (108), light yellow (32), green (1135), brown (1775), salmon (256), turquoise (2500), gray (2500), and blue (2500). These modules serve as clusters of related genes that exhibit coordinated expression patterns across the glioma grades, which can provide further insights into the underlying molecular foundations and regulatory networks in glioma.

### 3.2. Module Preservation Analysis

**Figure 4:**
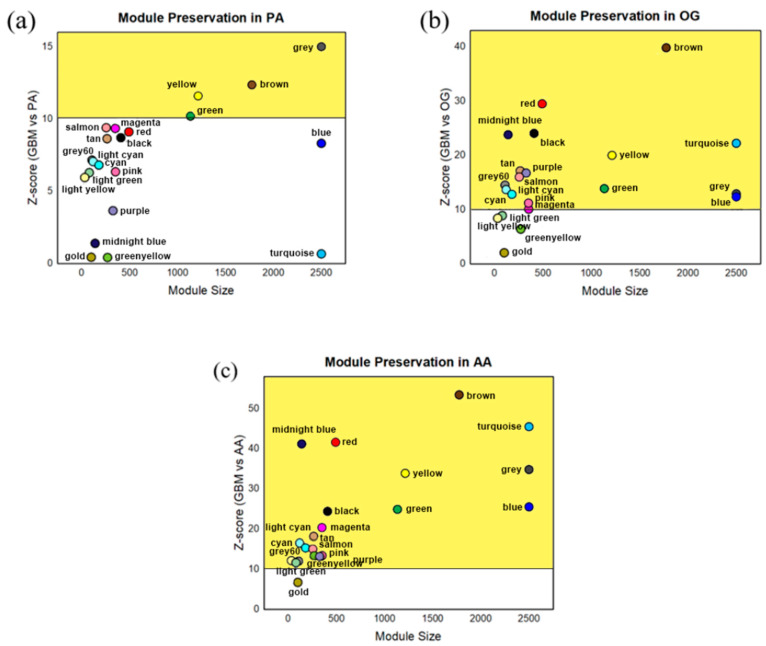
Module preservation analysis performed on the gene co-expression modules from the GBM network in (**a**) pilocytic astrocytoma, (**b**) oligodendroglioma, and (**c**) anaplastic astrocytoma datasets. The modules in the yellow partition pertain to highly preserved modules (Z > 10); green, yellow, brown, and gray exhibited high preservation in all datasets.

The preservation of the modules identified within the gene co-expression network of GBM was assessed in the datasets for PA, OG, and AA using the Z-summary score, which measures the connectivity of the modules that indicate the gain of preservation; a higher Z score indicated better preservation. As shown in Figure 4, there are few significant variations between the preserved modules across the datasets. In addition, most modules retain their relative position in the graphs but with more modules surpassing the threshold per dataset, as demonstrated by the increase in the Z-score values of the modules from the PA to the AA datasets. Biologically, this incidence is consistent with the severity of glioma, since the module eigengenes projected on the GBM dataset reflected increasing preservation from PA to AA, demonstrating the similarity of genes towards GBM. It may also be speculated that this relates to glioma progression. This results in the AA dataset having the highest number of significantly preserved modules, although only the modules with preservation scores higher than Z = 10 across all datasets—represented by the green, yellow, brown, and gray modules—were identified as modules of interest since the analysis specifically focused only on shared networks across the different glioma grades.

In addition, the analysis of module membership included the application of kME (eigengene-based connectivity) to measure the connectivity of each gene within a specific module. This was determined by calculating the correlation between the gene’s expression profile and the module eigengene, which functions as a representative expression profile for that module [26]. After that, the genes in each module were ranked according to their kME values in each dataset (Figure S2). The genes with the highest maximum rank across all datasets were chosen for the subsequent functional annotation and pathway enrichment analysis. Such genes consistently showed strong correlation within the particular modules across different glioma grades, indicating their critical roles in the biological processes that these modules represent.

### 3.3. Functional Annotation and Pathway Enrichment

**Figure 5:**
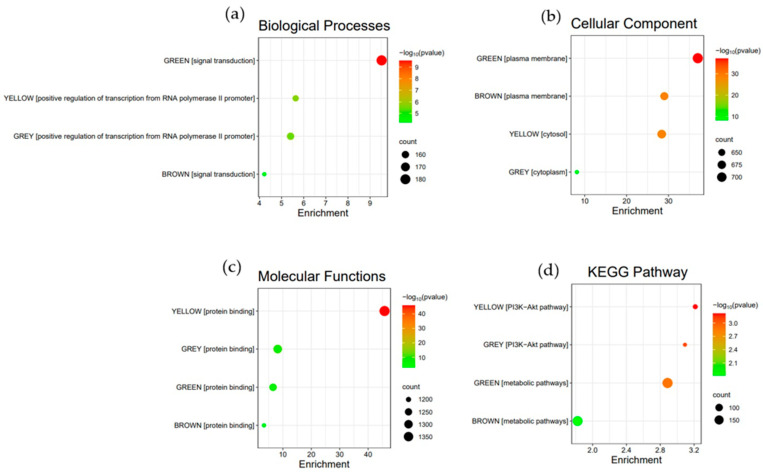
Top-enriched terms for the green, yellow, brown, and gray modules in terms of (**a**) biological processes, (**b**) cellular component, (**c**) molecular functions, and (**d**) KEGG pathways.

The DAVID webserver was used to cluster functional annotations based on the top genes in each module. For each module, the top-enriched Gene Ontology (GO) terms are shown in Figure 5a–c. Figure 5d shows KEGG pathways that were prioritized only if they were enriched in the same cluster as the top GO keywords. Significant enrichment scores were found in several clusters, which were noteworthy since they included links to the progression of glioma. Particularly, the green and brown modules were involved in changes in signaling pathways directly affecting metabolism, while the yellow and gray modules mainly affect transcription regulation. All modules were involved in protein binding. These findings may indicate that the green and brown modules are linked with the same or closely related metabolic pathways, as they both operate in the plasma membrane. On the other hand, pathway enrichment results have shown the involvement of the yellow and gray modules in the PI3K/Akt pathway, although possibly in different processes since the gray module could be operating in a specific cytoplasmic organelle as compared to the yellow module, which was indicated to be in the free cytoplasm.

### 3.4. Identification of Protein–Protein Interaction Networks and Hub Genes

**Figure 6:**
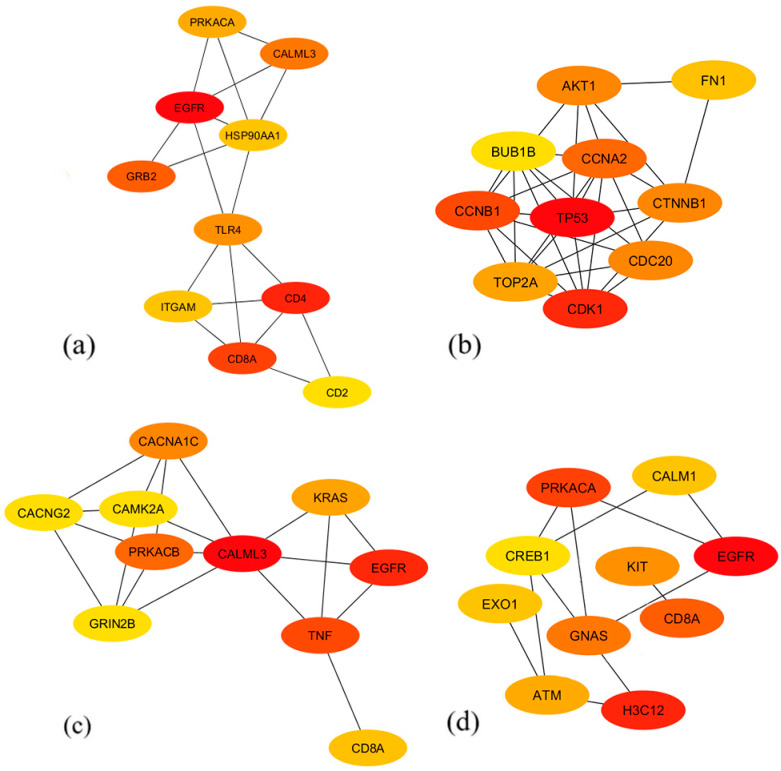
The identified top 10 hub gene networks based on the PPI networks of the (**a**) green, (**b**) yellow, (**c**) brown, and (**d**) gray modules. Nodes represent the hub genes, and edges represent interactions between connected nodes. Colors represent ranking with each network, from red (high) to yellow (low).

PPI networks were built for each module of interest using the STRING database to further investigate any possible connections between the different proteins corresponding to the genes within the discovered modules. To guarantee the accuracy of the anticipated interactions, a high confidence level of 0.7 was established [21]. In order to determine probable hub genes, each PPI network was imported into Cytoscape, and its network and gene (node) scores were calculated. In Cytoscape, CytoHubba was used to rank the genes within each network through the topological algorithm degree centrality, and the ten genes with the highest rankings for each network were considered as hub genes (Figure 6). The color of the genes represents the ranking of the hub genes, with the strongest red being the highest ranking. These identified hub genes require further analysis to determine their possible roles in the main pathways and processes associated with glioma due to their high interaction scores within the PPI networks of their corresponding modules, which indicates their crucial role in the gene network.

### 3.5. Signature-Based Drug Repurposing

In order to search for drugs that can reverse the expression of the top 10 hub genes from each module, transcriptional and molecular signatures were analyzed through the DRE webserver. Table 2 lists the top therapeutic candidates obtained together with the relevant mechanisms of action. Consideration was given to the top five potential drug candidates with the lowest false discovery rate (FDR) and Tau values, as compounds with low FDR tend to produce fewer false positive drug discovery results, and compounds with more negative Tau values induce more effective gene expression changes, countering the provided gene signature. For the upregulated hub genes, norgestimate, phentolamine, GW0742, olomoucine, and ambroxol were the top-ranked therapeutic candidates, whereas ethisterone, noscapine, nomegestrol, carmoxirole, and oxcarbazepine were the most promising drug candidates for the downregulated hub genes.

## 4. Discussion

### 4.1. Gene Co-Expression Modules across the Datasets

**Figure 7:**
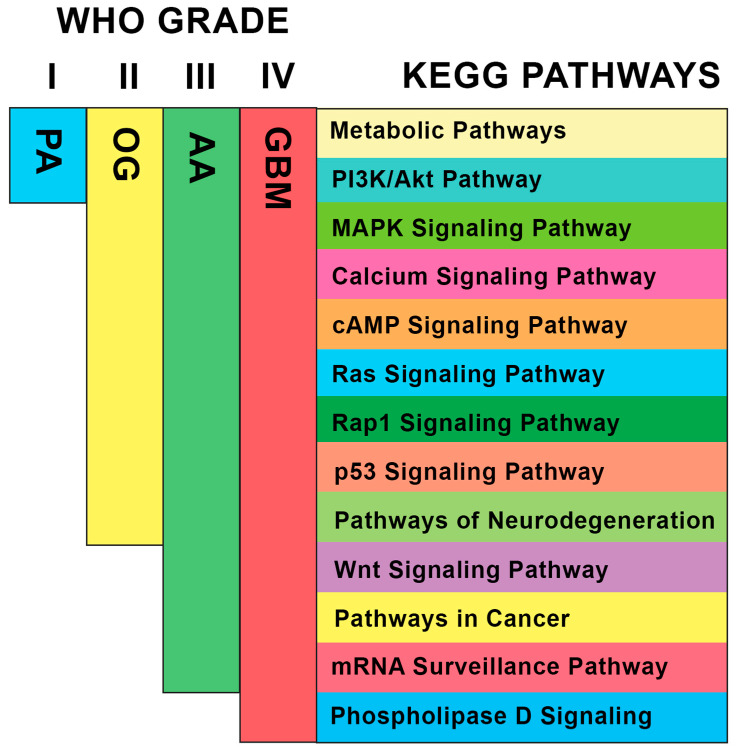
KEGG pathways of modules with increasing preservation per glioma grade (I, II, III, and IV) based on module preservation and pathway enrichment analysis results. Signaling pathways within the range of glioma grade are highly preserved (z > 10).

**Figure A3:**
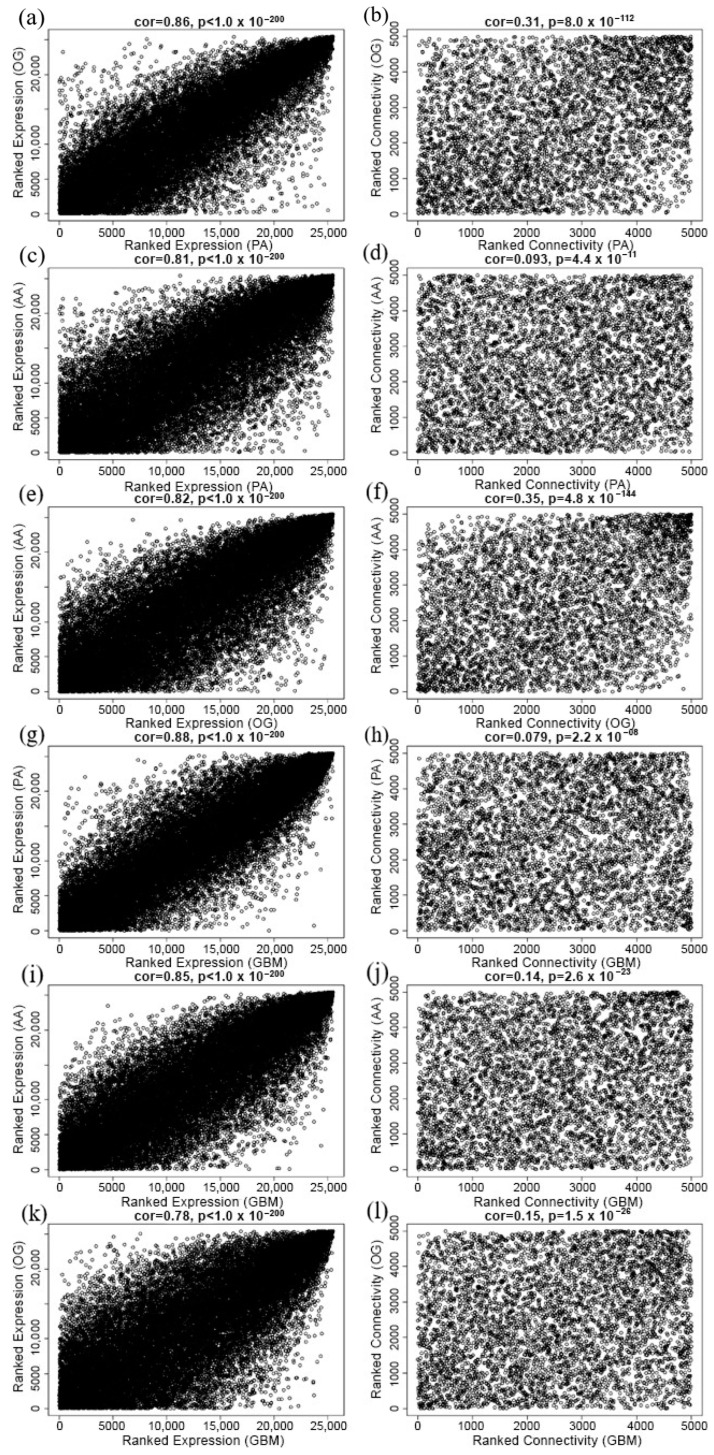
Ranked expression plots and ranked connectivity plots for dataset comparison: (**a**,**b**) PA vs. OG; (**c**,**d**) PA vs. AA; (**e**,**f**) OG vs. AA; (**g**,**h**) GBM vs. PA; (**i**,**j**) GBM vs. AA; and (**k**,**l**) GBM vs. OG.

The WHO categorized the different grades of glioma based on histological features, molecular characteristics, and clinical behaviors. The vast heterogeneity of gliomas per grade and cell origin is one of the significant factors contributing to its difficulty responding well to treatment [27]. Through WGCNA, this study focused on identifying points at which the distinct glioma grades align genetically, since several studies have demonstrated the effectiveness of this approach in determining highly correlated genes in related diseases from a systems biology point of view [28,29,30]. Furthermore, it is able to determine pathways involved in disease networks due to its ability to investigate the interplay among the highly preserved genes. These reasons supported the use of WGCNA to elaborate the disease network present in different glioma grades, represented by PA, OG, AA, and GBM datasets. Since the module eigengenes of other datasets were projected onto the GBM dataset, it can be speculated that the increasing trend in the module preservation analysis from the PA dataset to the AA dataset (Figure 4) was related to the fact that GBM is at the highest severity of the disease. This can also be observed in Figure A3, where the ranked connectivity of GBM across the datasets increases from the PA dataset to the AA dataset. With the recent discovery of the possible lineage conversion of oligodendroglioma to astrocytoma [31], the network’s relation to glioma progression was further investigated. Figure 7 shows the top KEGG pathways per module (Table A2) that may potentially serve as pivotal points of glioma progression, as it demonstrates the increasing preservation of modules per glioma grade with reference to GBM. It is important to note that the modules that did not reach the threshold of high preservation (z > 10) may still induce effects in other signaling pathways depending on the PPI networks. Particularly, the deregulation of the PI3K/Akt pathway is known to cause changes in MAPK/ERK, calcium signaling, and cAMP signaling pathways; Ras and Rap1 proteins are known regulators of the MAPK/ERK pathway [32,33,34,35,36]. On the other hand, metabolite biomarkers in neurodegenerative diseases have been discovered in recent studies, showing their connection to metabolic pathways [37,38]. Moving on, high preservation of the Wnt/β-catenin signaling pathway in AA suggests its tendency to migrate [39], and the deregulation of the mRNA surveillance pathway poses challenges in neurodegeneration [40]. Lastly, phosphatidic acid is known to regulate the PI3K/Akt signaling pathway through mTOR [41]. Hence, it can be hypothesized that there are existing synergistic processes, cross-talks, or even signal amplification events involved in these pathways, which may be relevant to glioma progression. This study may open new avenues for future explorations in glioma research for studies involving computational, wet laboratory, and clinical experimentations.

However, only the co-expression modules green, yellow, brown, and gray were highly preserved in all the datasets and subjected to further analysis. The functional annotation and pathway enrichment analysis was able to deduce that the green and brown modules are primarily associated with signal transduction deregulation in metabolic pathways, while the yellow and gray modules are mainly involved with cell proliferation of glioma, specifically the PI3K/Akt pathway (Table A3, Table A4, Table A5 and Table A6).

### 4.2. Module Hub Genes and Their Protein Functions

#### 4.2.1. Involvement of PI3K/Akt Pathway and Other Signaling Pathways

The hub genes of each highly preserved module have been identified and are listed along with their protein functions in Table A1. Notably, the *EGFR* gene was present across the green, brown, and gray modules while retaining its high interaction score, implying the strong involvement of the PI3K/Akt pathway, as implied by the pathway enrichment results. It has been seen as an important signaling pathway in the progression of low-grade to high-grade glioma since it is commonly upregulated through several types of genetic or post-translational mutations [42]. With this, many have made use of EGFR inhibitors, as most of the *EGFR* mutations in cancer cells cause overactivation of the PI3K-Akt pathway. However, the lack of blood–brain barrier permeability posed as its major obstacle [43,44]. Aside from *EGFR*, *AKT1* was also included in the upregulated hub genes, indicating the pathway’s relevance to the present study. Overexpression of the *AKT1* gene has been one of the common characteristics of glioma cells, as it promotes viability and malignancy [45]. On the other hand, the *PRKACA* and *PRKACB* genes that may indirectly inhibit the activation of the PI3K/Akt pathway through the cAMP signaling pathway were downregulated. This is consistent with the need to prevent apoptosis and ensure the survival of the gliomas.

The inclusion of hub genes primarily involved in other signaling pathways that affect the PI3K/Akt pathway further supports the existing pathway interconnectivity in glioma. It was found that *KRAS* and *GRB2* in the MAPK/ERK signaling pathway were upregulated across the datasets. Recent studies have indicated the strong cooperativity between the PI3K/AKT and MAPK/ERK signaling pathways in glioma progression due to them being regulators of cell proliferation [32,34]. Aside from this, the *CTNNB1* and *FN1* genes in the Wnt/β-catenin signaling pathway were also upregulated. The relationship between the Wnt/β-catenin and PI3K/Akt signaling pathways has been known to induce glioma malignancy [39]. Lastly, it was observed that the calcium signaling pathway is significantly involved, represented by the *CACNA1C*, *CACNG2*, *CALML3*, *CALM1*, and *CAMK2A* hub genes. Its dysregulation affects the PI3K/Akt pathway by increasing calcium influx, which further activates biological processes such as growth, gene expression, and neurotransmitter release, and it is claimed that the calcium signaling pathway is another relevant factor contributing to the motility and invasion of gliomas [33,46].

#### 4.2.2. Deregulation of Cellular Processes in Glioma

**Figure 8:**
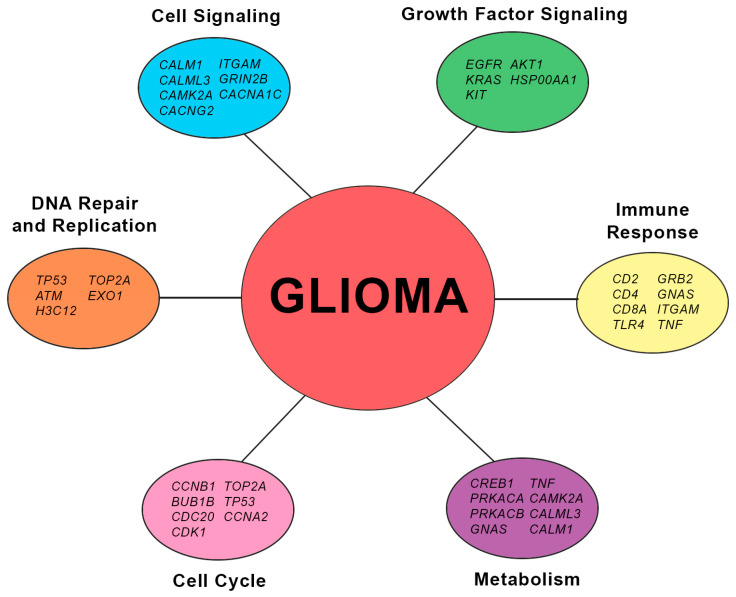
Cellular processes interconnectivity based on hub gene functions.

Several upregulated hub genes relating to DNA repair have been observed in the gray module, such as *EXO1*, *H3C12*, and *ATM*. In cancer cells, the upregulation of genes involved in DNA repair may indicate increased DNA damage that may possibly be due to genomic instability, increased replication stress, and resistance to DNA-damaging therapies [47]. Specifically, high expression of *ATM* gene has been shown to be linked with radio-resistance of glioma [48]. On the other hand, upregulated hub genes in the yellow module partake mainly on cell cycle regulation. The *TP53* gene, which encodes for the p53 protein, which is a main regulator of both the cell cycle and DNA repair and replication, was found to be upregulated across the datasets. It functions by inducing the expression of DNA repair genes while inhibiting cell cycle progression in response to DNA damage to ensure genomic integrity, although cancer cells normally observe upregulated expression of *TP53* due to mutations that enable them to become chemo-resistant [49]. In general, the upregulation of genes involved in the cell cycle may promote cell cycle progression, genomic instability, and enhanced proliferation, which could have an impact on the behavior of tumors and their response to therapeutics. Across the modules, hub genes that are involved in immune response were also observed. Among these, genes that code for clusters of differentiation proteins such as *CD4*, *CD2*, and *CD8A* were found to be downregulated and observed in the green module, while CD8A was also present in the brown and gray modules. The downregulation of these genes may indicate the impairment of immune surveillance and effector functions of T cells and natural killer cells that overall modulate immune responses against anomalies, including tumors. Figure 8 shows a graphical summary of the hub genes and the potential cellular processes affected due to their involvement in glioma. Furthermore, the involvement of specific hub genes in several cellular processes supports the existing pathway interconnectivity.

### 4.3. Metabolic Reprogramming of Glioma Cells

Collectively, the presented results indicate that the gene network constructed is primarily involved with metabolic reprogramming of glioma, as the modules were found to be concerned with the PI3K/Akt and metabolic pathways. Metabolic reprogramming is a major hallmark in glioma, as it is when tumor cells alter their metabolism into aerobic glycolysis to generate the necessary energy to invade surrounding cells and adapt to different microenvironments [50,51]. Aside from metabolizing glucose for energy production, cancer cells are able to utilize lipids as raw materials for energetic currency [52]. In particular, the green and brown modules were highly involved with metabolic pathways and operate in cytosol, which aligns with malignant glioma cells also being dependent on fatty acid oxidation [53,54]. Furthermore, these metabolic changes are known to be regulated by phosphoinositide 3-kinase/protein kinase (PI3Ks), the main ligand for the PI3K/Akt pathway, as it can directly regulate the TCA cycle, wherein increased PI3K activation also increases AKT activity, which promotes citrate conversion to acetyl-CoA for fatty acid synthesis [51].

### 4.4. Signature-Based Drug Repurposing

The involvement of several signaling pathways affecting the PI3K/Akt pathway creates avenues into which therapeutics may be introduced. Through the DRE webserver, potential drug candidates were screened, as shown in Table 3, along with their corresponding pathways and affected processes based on their mechanism of action. Norgestimate, ethisterone, and nomegestrol are synthetic progestin hormones that mimic the effects of progesterone to activate physiological responses through binding with progesterone receptors [55,56,57]. The influence of sex hormones on glioma progression was observed in women, who are otherwise less likely to develop glioma than men but who become more prone to developing glioma after menopause due to decreased estrogen production [58,59,60]. Progesterone, on the other hand, can indirectly modulate estrogen production by interfering with estrogen receptor activity or releasing gonadotropins through feedback mechanisms [61]. Despite producing pro-tumorigenic effects at low doses, high doses of progesterone have demonstrated therapeutic effects against GBM by downregulating the activity of progesterone receptor B (PR-B), a protein that promotes tumor cell growth, or through alteration of detoxification mechanisms, stress, immune response, and glucose metabolism [62,63,64,65,66,67]. Furthermore, a significant decrease in the expression levels of EGFR, Akt, phospho-Akt, mTOR, and phospho-mTOR has been observed following high doses of progesterone, indicating its potential attenuating effect against GBM through the PI3K/Akt and metabolic pathways [68,69,70]. Aside from this, progesterone has a known regulatory effect in the significant signaling pathways previously mentioned, in cAMP, MAPK/ERK, Wnt/β-catenin, and calcium signaling pathway in other types of cancer [71,72,73,74], which further demonstrates that the use of synthetic progestin hormones against glioma progression can be a potential area of interest.

Phentolamine, an adrenergic receptor antagonist drug, also scored significantly, along with other approved and experimental drugs, as shown in Table 2. Previous studies have shown the regulating effect of adrenergic receptor antagonist drugs against cancer initiation and progression due to their influence on the cAMP/PKA, MAPK/ERK, and PI3K/Akt pathways [75,76]. Another drug candidate is GW0742, as its potential modulating effect towards fatty acid oxidation has become an area of interest for cancer treatment research [77,78]. A CDK inhibitor, olomoucine, was also included as a potential drug candidate due to its regulation of the cell cycle. CDK inhibitors promote cell cycle arrest and apoptosis and decrease cell proliferation in glioma [79]. Ambroxol and oxcarbazepine were found to be possible drug candidates due to their disrupting effect on the depolarization process and ion homeostasis, in which calcium signaling is also affected [80]. On the other hand, noscapine, a bradykinin receptor antagonist, also interferes with the calcium signaling pathway by reducing calcium production and, subsequently, glioma cell migration [81]. Lastly, a dopamine receptor agonist, carmoxirole, was included due to its potential to modulate cAMP production, with dopamine receptor D1 inhibiting GBM tumorigenicity and dopamine receptor D2 modulating GBM survival and death [82,83].

## 5. Conclusions

The present study was able to successfully determine the four highly conserved modules in different glioma grades that were represented by PA (GSE50161), OG (GSE4290), AA (GSE43378), and GBM (GSE36245). Through WGCNA, the top 10 hub genes per module were identified and used to screen for potential drug candidates. Furthermore, the functional annotation and pathway enrichment analysis paved the way for deducing the complex molecular basis for glioma, focusing on the PI3K/Akt pathway, signal transduction deregulation, and metabolic reprogramming. Based on the hub genes, several signaling pathways associated with the PI3K/Akt pathway, such as the cAMP signaling pathway, MAPK/ERK, Wnt/β-catenin, and calcium signaling, have also been deregulated, which often results in metabolic reprogramming. The involvement of several signaling pathways in deregulating the PI3K/Akt pathway and vice versa further indicates the possible interconnection of signaling pathways in glioma severity. The synthetic progestin hormones norgestimate and ethisterone were the top drug candidates identified in the transcriptional signature-based drug repurposing approach. Aside from these, several experimental and approved drug candidates have also been identified, including an adrenergic receptor antagonist, a PPAR-γ receptor agonist, a CDK inhibitor, a sodium channel blocker, and a bradykinin receptor antagonist, which further demonstrates that the gene network is a potential therapeutic avenue for glioma. Our study posts limitations since all were conducted in silico; however, the theoretical data presented can be used as a platform to explore avenues in designing drug cocktails or drug candidates for glioma. Furthermore, this study provides insights on the interconnectivities of molecular signaling, which can be further evaluated using wet laboratory experimentations.

## Figures and Tables

**Table 1:** Summary of GEO Datasets.

Accession No.	GSE50161	GSE4290	GSE43378	GSE36245
Condition	Pilocytic Astrocytoma	Oligodendroglioma	Anaplastic Astrocytoma	Glioblastoma Multiforme
Type	Expression Profiling by Array			
Platform	GPL570-HG-U133 Plus 2 Affymetrix Human Genome U133 Plus 2.0 Array			
Source	Primary Brain Tumor Samples			
No. of Samples	14	36	19	44

**Table 2:** Top five drug candidates for the upregulated and downregulated hub genes.

Expression	Genes	Drug	Mechanism	Tau	FDR
Upregulated	KRAS, CCNB1, BUB1B, KIT, TP53, EGFR, ATM, EXO1, GNAS, CDC20, TOP2A, HSP90AA1, FN1, H3C12, GRIN2B, GRB2, CCNA2, CDK1, CALM1, CALML3, CAMK2A, CREB1, TNF, AKT1, CTNNB1, and ITGAM	Norgestimate Phentolamine GW0742 Olomoucine Ambroxol	Progesterone receptor agonist Adrenergic receptor antagonist PPAR receptor agonist CDK inhibitor Sodium channel blocker	−99.8 −99.7 −99.5 −99.4 −99.3	7.75 × 10^−3^ 6.53 × 10^−3^ 5.05 × 10^−4^ 7.01 × 10^−3^ 8.35 × 10^−3^
Downregulated	CD4, CACNA1C, CACNG2, CD2, PRKACA, PRKACB, TLR4, and CD8A	Ethisterone Noscapine Nomegestrol Carmoxirole Oxcarbazepine	Progestogen hormone Bradykinin receptor antagonist Progestogen hormone Dopamine receptor agonist Sodium channel blocker	−99.7 −99.6 −99.5 −99.5 −99.5	2.36 × 10^−3^ 6.22 × 10^−5^ 7.89 × 10^−4^ 7.40 × 10^−3^ 9.94 × 10^−3^

**Table 3:** Potential drug candidates and their corresponding pathways or processes impeded.

Drug	Status	Pathway/Process	Reference
Norgestimate	Approved	PI3K/Akt Pathway	[68,69,70]
Phentolamine	Approved	cAMP Signaling, MAPK/ERK, and PI3K/Akt Pathway	[75,76]
GW0742	Experimental	Lipid Metabolic Pathways	[77,78]
Olomoucine	Approved	Cell Cycle	[79]
Ambroxol	Approved	Calcium Signaling Pathway	[80]
Ethisterone	Approved	PI3K/Akt Pathway	[68,69,70]
Noscapine	Approved	Calcium Signaling Pathway	[81]
Nomegestrol	Approved	PI3K/Akt Pathway	[68,69,70]
Carmoxirole	Experimental	cAMP Signaling Pathway	[82,83]
Oxcarbazepine	Approved	Calcium Signaling Pathway	[80]

## Data Availability

The gene microarray datasets used for the study are openly available in the NCBI Gene Expression Omnibus (GEO) database under the accession IDs GSE50161, GSE4290, GSE43378, and GSE36245 at https://www.ncbi.nlm.nih.gov/geo/ accessed on 7 December 2023.

## Supplementary Materials

The following supporting information can be downloaded at www.mdpi.com/article/10.3390/biology13040206/s1: Figure S1: TOM-based dissimilarity clustering of (a) PA, (b) OG, (c) AA, and (d) GBM datasets; Figure S2: Summary of in-module connectivities of genes in highly preserved modules across all datasets referenced in GBM and plotted against (a) PA, (b) OG, and (c) AA datasets.

## Appendix A

**Table A1:** Summary of module hub genes and their protein functions obtained from the GeneCards database (www.genecards.org (accessed 27 December 2023)) [84].

Gene	Protein	Function	Module/s
EGFR	epidermal growth factor receptor	activates MAPK and PI3K/AKT pathways; promotes cell proliferation and survival	Green, brown, and grey
CD8A	CD8 subunit alpha	binds to MHC class I; facilitates T cell-mediated cytotoxicity against infected or transformed cells	Green, brown, and grey
CALML3	calmodulin like 3	regulates calmodulin-dependent signaling; potential roles in intracellular calcium homeostasis	Green and brown
PRKACA	protein kinase cAMP-activated catalytic subunit alpha	phosphorylates target proteins; involved in cAMP signaling; regulates various cellular processes	Green and grey
CACNA1C	calcium voltage-gated channel subunit alpha1 C	mediates calcium ion influx; regulates neuronal excitability and cardiac function	Brown
CAMK2A	calcium/calmodulin dependent protein kinase II alpha	regulates synaptic plasticity and memory formation	Brown
PRKACB	protein kinase cAMP-activated catalytic subunit beta	involved in cAMP signaling; regulates various cellular processes	Brown
CACNG2	calcium voltage-gated channel auxiliary subunit gamma 2	modulates channel function; regulates calcium ion influx and neuronal excitability	Brown
KRAS	KRAS proto-oncogene, GTPase	activates MAPK pathway; regulates cell proliferation, survival, and differentiation	Brown
TNF	tumor necrosis factor	regulates inflammation, apoptosis, and immune cell functions	Brown
GRIN2B	glutamate ionotropic receptor NMDA type subunit 2B	mediates synaptic transmission; regulates synaptic plasticity, learning, and memory	Brown
ITGAM	integrin subunit alpha M	regulates immune cell adhesion, migration, and phagocytosis	Green
HSP90AA1	heat shock protein 90 alpha family class A member 1	assists in protein folding and stabilization	Green
GRB2	growth factor receptor bound protein 2	links receptor tyrosine kinases to Ras/MAPK pathway activation	Green
TLR4	Toll-like receptor 4	detects lipopolysaccharides; activates innate immune responses	Green
CD4	CD4 molecule	binds to MHC class II; facilitates antigen recognition and T cell activation	Green
CD2	CD2 molecule	regulates immune cell interactions; facilitates T cell adhesion and co-stimulation	Green
CALM1	calmodulin 1	modulates calcium-dependent signaling pathways; regulates cellular responses to calcium ions	Grey
EXO1	exonuclease 1	involved in DNA mismatch repair and recombination; maintains genomic stability	Grey
KIT	KIT proto-oncogene, receptor tyrosine kinase	activates PI3K/AKT and MAPK pathways; regulates cell proliferation, survival, and differentiation	Grey
CREB1	cAMP responsive element binding protein 1	binds to cAMP response elements; regulates gene expression in response to cAMP signaling	Grey
GNAS	G-protein alpha subunit	activates adenylyl cyclase; regulates cellular signaling pathways via GPCRs	Grey
ATM	ATM serine/threonine kinase	regulates DNA damage response; activates cell cycle checkpoints and DNA repair mechanisms	Grey
H3C12	H3 clustered histone 12	packaging and organizing of DNA into nucleosome	Grey
FN1	fibronectin 1	mediates cell adhesion and migration; regulates tissue remodeling and repair	Yellow
AKT1	AKT serine/threonine kinase 1	activates mTOR and other downstream pathways; regulates cell survival, growth, and metabolism	Yellow
BUB1B	BUB1 mitotic checkpoint serine/threonine kinase B	regulates chromosome alignment and segregation; ensures genomic stability during cell division	Yellow
CCNA2	cyclin A2	forms complexes with CDKs; regulates G1/S and G2/M transitions of the cell cycle	Yellow
CTNNB1	catenin beta 1	component of adherens junctions; regulates cell-cell adhesion and Wnt signaling pathway	Yellow
CDC20	cell division cycle 20	facilitates ubiquitination and degradation of cell cycle regulators; controls mitotic progression	Yellow
TP53	tumor protein p53	activates DNA repair or apoptosis in response to DNA damage; regulates cell cycle checkpoints	Yellow
CCNB1	cyclin B1	forms complexes with CDK1; regulates the G2/M transition of the cell cycle	Yellow
TOP2A	DNA topoisomerase II alpha	regulates DNA topology during replication and transcription; targeted in cancer therapy	Yellow
CDK1	cyclin dependent kinase 1	forms complexes with cyclins; regulates cell cycle transitions and mitotic entry	Yellow

**Table A2:** Top KEGG pathway analysis results in identified modules obtained from the Kyoto Encyclopedia of Genes and Genomes (KEGG) database (https://www.genome.jp/kegg (accessed 27 December 2023)) [20].

Module	Term	Count	Adj. *p*-Value
Green	hsa01100 metabolic pathways	194	1.4 × 10^−3^
Brown	hsa01100 metabolic pathways	172	1.3 × 10^−2^
Yellow	hsa04151 PI3K/Akt signaling pathway	59	6.3 × 10^−4^
Gray	hsa04151 PI3K/Akt signaling pathway	56	2.9 × 10^−3^
Purple	hsa04020 calcium signaling pathway	65	2.8 × 10^−13^
Tan	hsa04015 Rap1 signaling pathway	29	2.0 × 10^−2^
Cyan	hsa04020 calcium signaling pathway	51	3.6 × 10^−7^
Black	hsa04020 calcium signaling pathway	55	3.9 × 10^−9^
Magenta	hsa04014 Ras signaling pathway	34	9.2 × 10^−4^
Pink	hsa04024 cAMP signaling pathway	45	8.1 × 10^−6^
Salmon	hsa04010 MAPK signaling pathway	52	6.1 × 10^−6^
Turquoise	hsa04010 MAPK signaling pathway	38	1.3 × 10^−2^
Blue	hsa04020 calcium signaling pathway	68	1.9 × 10^−10^
Gray60	hsa04010 MAPK signaling pathway	53	1.2 × 10^−5^
Midnight Blue	hsa04151 PI3K/Akt signaling pathway	51	4.2 × 10^−3^
Red	hsa04115 p53 signaling pathway	21	1.4 × 10^−5^
Light Cyan	hsa05022 pathways of neurodegeneration-multiple diseases	59	2.3 × 10^−2^
Green-yellow	hsa03015 mRNA surveillance pathway	31	8.8 × 10^−9^
Light Yellow	hsa04310 Wnt signaling pathway	25	9.8 × 10^−3^
Light Green	hsa05200 pathways in cancer	75	1.2 × 10^−3^
Gold	hsa04072 phospholipase D signaling pathway	26	5.1 × 10^−3^

**Table A3:** Top GO and pathway analysis results in brown module obtained from the Gene Ontology Consortium (http://www.geneontology.org (accessed 27 December 2023)) and the Kyoto Encyclopedia of Genes and Genomes (KEGG) database (https://www.genome.jp/kegg (accessed 27 December 2023)) [20,85].

Category	Term	Count	Adj. *p*-Value
BP	GO:0007165 signal transduction	150	7.8 × 10^−5^
	GO:0035556 intracellular signal transduction	69	4.4 × 10^−6^
	GO:0006468 protein phosphorylation	64	3.1 × 10^−5^
	GO:0006508 proteolysis	62	2.5 × 10^−4^
	GO:0007268 chemical synaptic transmission	56	9.4 × 10^−11^
CC	GO:0005886 plasma membrane	667	3.8 × 10^−29^
	GO:0016020 integral component of membrane	588	4.4 × 10^−12^
	GO:0005737 cytoplasm	559	9.7 × 10^−5^
	GO:0005829 cytosol	543	2.0 × 10^−4^
	GO:0016020 membrane	424	1.9 × 10^−12^
MF	GO:0005515 protein binding	1192	5.4 × 10^−4^
	GO:0005524 ATP binding	180	1.1 × 10^−4^
	GO:0042802 identical protein binding	174	6.4 × 10^−2^
	GO:0005509 calcium ion binding	105	5.3 × 10^−6^
	GO:0004712 protein serine/threonine/tyrosine kinase activity	68	1.4 × 10^−5^
KEGG	hsa01100 metabolic pathways	172	1.3 × 10^−2^
	hsa05200 pathways in cancer	68	9.3 × 10^−3^
	hsa05022 pathways of neurodegeneration-multiple diseases	60	2.0 × 10^−2^
	hsa04020 calcium signaling pathway	49	2.1 × 10^−6^
	hsa04010 MAPK signaling pathway	48	4.3 × 10^−4^

**Table A4:** Top GO and pathway analysis results in green module obtained from the Gene Ontology Consortium (http://www.geneontology.org (accessed 27 December 2023)) and the Kyoto Encyclopedia of Genes and Genomes (KEGG) database (https://www.genome.jp/kegg (accessed 27 December 2023)) [20,85].

Category	Term	Count	Adj. *p*-Value
BP	GO:0007165 signal transduction	181	4.7 × 10^−10^
	GO:0030154 cell differentiation	89	1.1 × 10^−3^
	GO:0007155 cell adhesion	87	3.6 × 10^−7^
	GO:0045087 innate immune response	83	2.6 × 10^−4^
	GO:0006915 apoptotic process	75	4.2 × 10^−3^
CC	GO:0005886 plasma membrane	719	1.0 × 10^−37^
	GO:0005737 cytoplasm	630	4.9 × 10^−11^
	GO:0016020 integral component of membrane	595	3.3 × 10^−10^
	GO:0005829 cytosol	569	4.2 × 10^−5^
	GO:0016020 membrane	431	2.6 × 10^−11^
MF	GO:0005515 protein binding	1256	3.3 × 10^−7^
	GO:0042802 identical protein binding	214	4.2 × 10^−6^
	GO:0005524 ATP binding	172	4.2 × 10^−3^
	GO:0005509 calcium ion binding	118	1.0 × 10^−8^
	GO:0042803 protein homodimerization activity	93	2.3 × 10^−3^
KEGG	hsa01100 metabolic pathways	194	1.4 × 10^−3^
	hsa05200 pathways in cancer	82	1.6 × 10^−4^
	hsa04010 MAPK signaling	56	1.5 × 10^−5^
	hsa04020 calcium signaling	53	7.3 × 10^−7^
	hsa04151 PI3K/Akt signaling pathway	51	1.6 × 10^−2^

**Table A5:** Top GO and pathway analysis results in gray module obtained from the Gene Ontology Consortium (http://www.geneontology.org (accessed 27 December 2023)) and the Kyoto Encyclopedia of Genes and Genomes (KEGG) database (https://www.genome.jp/kegg (accessed 27 December 2023)) [20,85].

Category	Term	Count	Adj. *p*-Value
BP	GO:0045944 positive regulation of transcription by RNA polymerase II	160	3.9 × 10^−6^
	GO:0007165 signal transduction	158	2.0 × 10^−4^
	GO:0000122 negative regulation of transcription by RNA polymerase II	116	9.2 × 10^−3^
	GO:0045893 positive regulation of DNA-templated transcription	97	1.6 × 10^−4^
	GO:0007155 cell adhesion	85	5.9 × 10^−6^
CC	GO:0005737 cytoplasm	628	7.0 × 10^−9^
	GO:0005886 plasma membrane	619	5.8 × 10^−12^
	GO:0005829 cytosol	596	1.3 × 10^−6^
	GO:0005634 nucleus	588	2.7 × 10^−2^
	GO:0016020 integral component of membrane	521	6.2 × 10^−2^
MF	GO:0005515 protein binding	1307	8.6 × 10^−9^
	GO:0046872 metal ion binding	288	2.3 × 10^−2^
	GO:0005524 ATP binding	217	1.1 × 10^−9^
	GO:0005509 calcium ion binding	110	5.5 × 10^−6^
	GO:0004712 protein serine/threonine/tyrosine kinase activity	78	1.3 × 10^−7^
KEGG	hsa04151 PI3K/Akt signaling pathway	56	2.9 × 10^−3^
	hsa04010 MAPK signaling pathway	49	7.3 × 10^−4^
	hsa04020 calcium signaling pathway	48	2.0 × 10^−5^
	hsa04015 Rap1 signaling pathway	38	4.4 × 10^−4^
	hsa04024 cAMP signaling pathway	36	5.5 × 10^−3^

**Table A6:** Top GO and pathway analysis results in yellow module obtained from the Gene Ontology Consortium (http://www.geneontology.org (accessed 27 December 2023)) and the Kyoto Encyclopedia of Genes and Genomes (KEGG) database (https://www.genome.jp/kegg (accessed 27 December 2023)) [20,85].

Category	Term	Count	Adj. *p*-Value
BP	GO:0045944 positive regulation of transcription by RNA polymerase II	155	2.3 × 10^−6^
	GO:0000122 negative regulation of transcription by RNA polymerase II	120	4.8 × 10^−4^
	GO:0045893 positive regulation of DNA-templated transcription	103	9.5 × 10^−7^
	GO:0006355 regulation of DNA-templated transcription	103	6.9 × 10^−2^
	GO:0006915 apoptotic process	99	4.0 × 10^−9^
CC	GO:0005829 cytosol	681	3.1 × 10^−29^
	GO:0005634 nucleus	679	7.3 × 10^−19^
	GO:0005737 cytoplasm	623	1.5 × 10^−13^
	GO:0005654 nucleoplasm	554	8.4 × 10^−36^
	GO:0016020 membrane	475	1.1 × 10^−24^
MF	GO:0005515 protein binding	1396	3.1 × 10^−46^
	GO:0046872 metal ion binding	268	7.0 × 10^−2^
	GO:0005524 ATP binding	232	1.3 × 10^−15^
	GO:0042802 identical protein binding	227	4.2 × 10^−9^
	GO:0003723 RNA binding	223	5.1 × 10^−15^
KEGG	hsa04151 PI3K/Akt signaling pathway	59	6.3 × 10^−4^
	hsa05200 pathways in cancer	53	2.7 × 10^−3^
	hsa04141 protein processing in endoplasmic reticulum	48	3.1 × 10^−10^
	hsa05205 proteoglycans in cancer	44	7.7 × 10^−6^
	hsa04010 MAPK signaling pathway	44	2.5 × 10^−2^
