# Supplementary material for: Modular Hub Genes in DNA Microarray Suggest Potential Signaling Pathway Interconnectivity in Various Glioma Grades
Source: Biology (Basel). 2024 Mar 23;13(4):206. doi: 10.3390/biology13040206 (PMC11048586; doi:10.3390/biology13040206)
Supplement: Supplementary file 1 [file biology-13-00206-s001.zip › biology-2892264-supplementary.pdf]

Supplementary Materials

Modular Hub Genes in DNA Microarray Suggests Potential Signaling Pathway Interconnectivity in Various Glioma Grades

Marco A. Orda, Peter Matthew T. Fowler, and Lemmuel L. Tayo

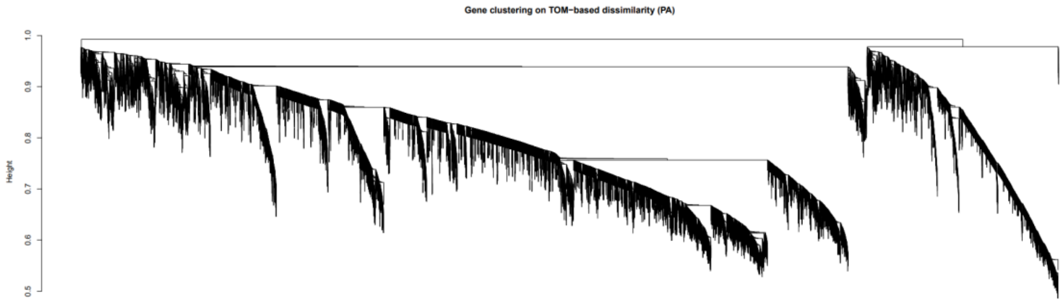

(a)

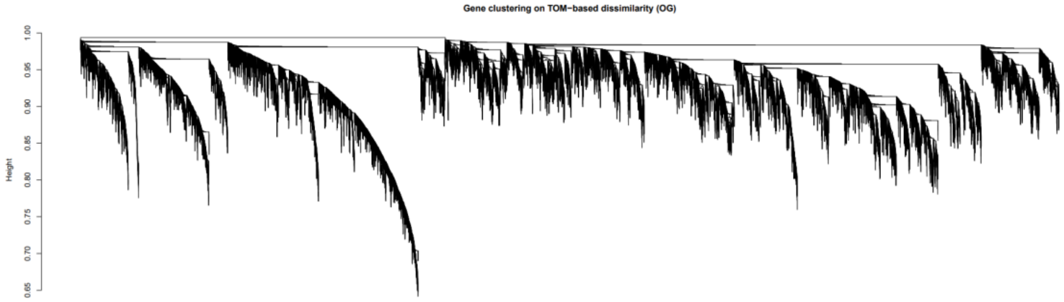

(b)

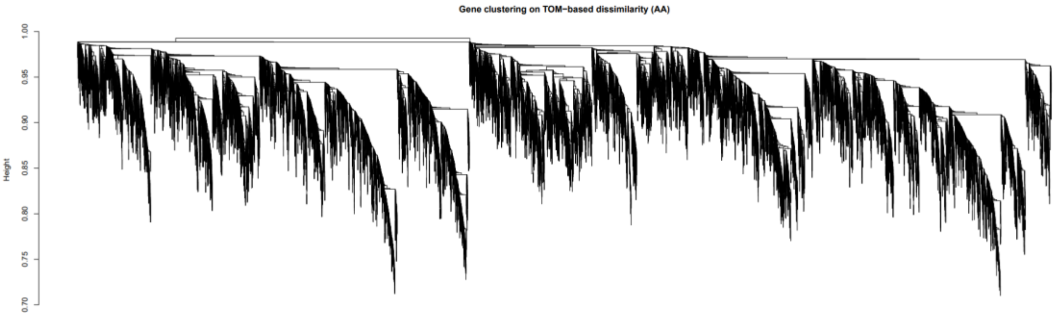

(c)

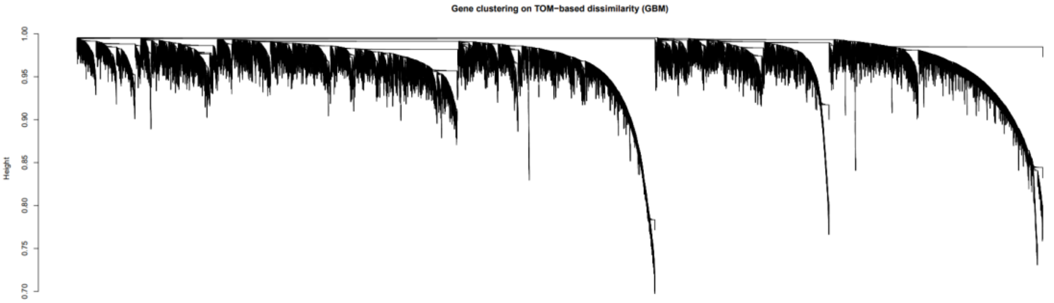

(d)

Figure S1. TOM-based dissimilarity clustering of (a) PA, (b) OG, (c) AA, and (d) GBM datasets.

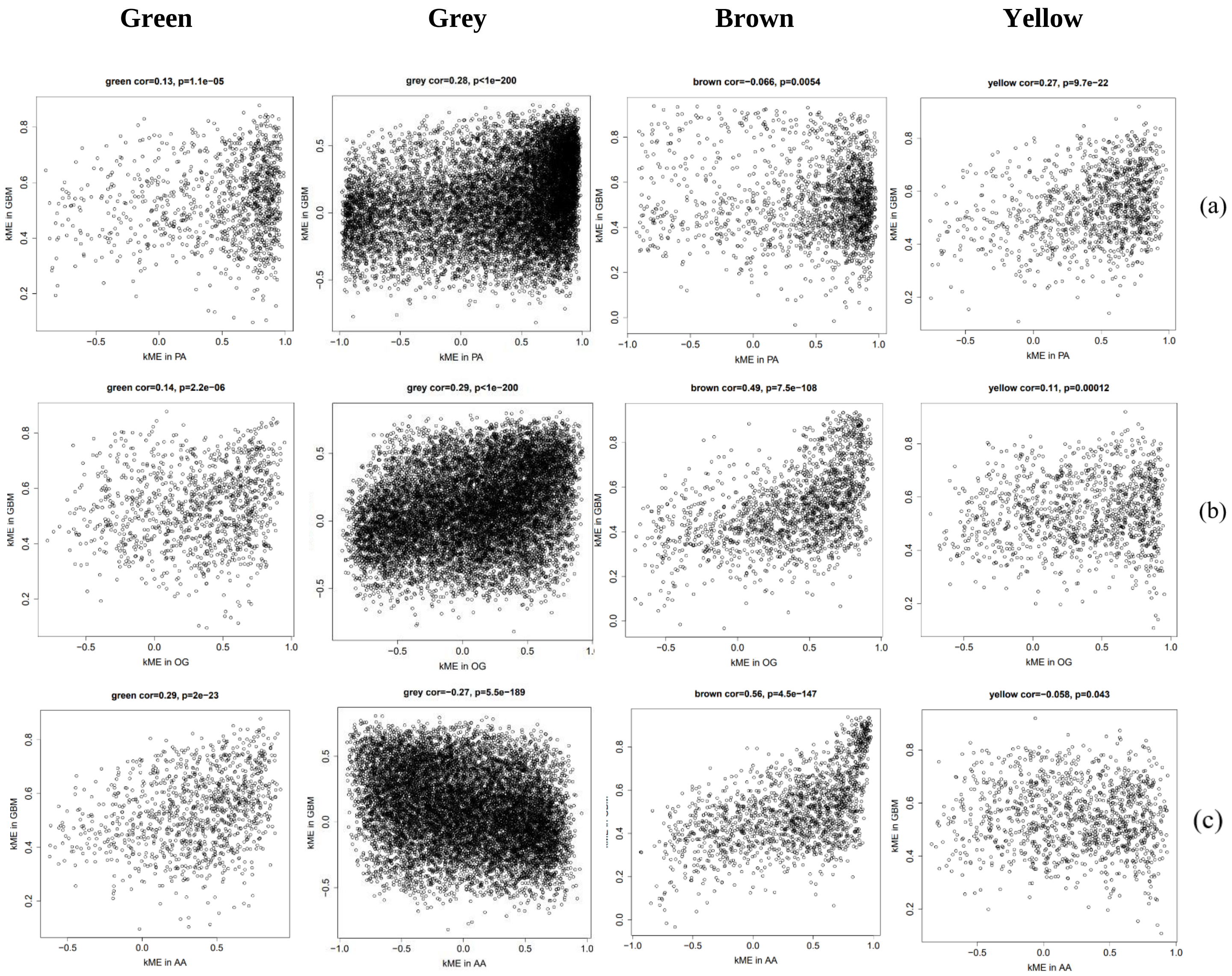

Figure S2. Summary of in-module connectivities of genes in highly preserved modules across all datasets referenced in GBM and plotted against (a) PA, (b) OG, and (c) AA datasets.
